# Murine Pancreatic Beta TC3 Cells Show Greater 2′, 5′-Oligoadenylate Synthetase (2′5′AS) Antiviral Enzyme Activity and Apoptosis Following IFN-*α* or Poly(I:C) Treatment than Pancreatic Alpha TC3 Cells

**DOI:** 10.1155/2009/631026

**Published:** 2009-10-29

**Authors:** M. Li, D. -J. Zheng, L. L. Field, V. Bonnevie-Nielsen

**Affiliations:** ^1^Department of Pathology and Laboratory Medicine, Child and Family Research Institute, University of British Columbia, 950 W 28th Ave., Vancouver, BC, Canada V5Z 4H4; ^2^Department of Medical Genetics, Child and Family Research Institute, University of British Columbia, 950 W 28th Ave., Vancouver, BC, Canada V5Z 4H4

## Abstract

Type 1 diabetes is caused by autoimmune destruction of pancreatic beta cells, possibly virus initiated. Virus infection induces alpha-interferon (IFN-*α*), leading to upregulation of genes encoding double-stranded (ds) RNA-dependent antiviral enzymes 2′, 5′-oligoadenylate synthetase (2′5′AS) and PKR (p68). To investigate whether beta cell specificity could be due to antiviral differences between beta and alpha cells, we treated beta and alpha TC3 cell lines with IFN-*α* and/or poly(I:C) (a synthetic dsRNA). Results showed that, following IFN-*α* stimulation, increases in 2′5′AS levels and activities were significantly higher in beta than alpha cells (*P* < .001), whereas increases in PKR level and activity were comparable in the two cell types. Poly(I:C) stimulated 2′5′AS activity in beta but not alpha cells, and co-transfection IFN-*α* plus poly(I:C) induced apoptosis in beta but not alpha cells. These findings suggest that the elevated 2′5′AS response of pancreatic beta cells could render them particularly vulnerable to damage and/or apoptosis during virus infection.

## 1. Introduction

Type 1 diabetes is a common and multifactorial disease characterized by autoimmune-mediated destruction of the insulin-producing beta cells of the pancreas. Both genetic and environmental factors play significant roles in the etiology of type 1 diabetes. Considerable evidence suggests that enterovirus infection, especially group B coxsackieviruses, is an important environmental factor initiating or promoting beta cell damage during the development of type 1 diabetes. A series of prospective Finnish studies have shown that there is a strong association between enterovirus infection and beta cell autoimmunity [1–4]. Increased frequencies of serum enterovirus antigens and antibodies against enterovirus were observed during the preclinical phase in children who subsequently developed diabetes [1, 3, 4]. Similarly, it was found that there was a temporal association between appearance of antiviral antibodies and islet cell autoantibodies in prediabetic siblings of type 1 diabetic patients [2, 4]. Furthermore, the finding of increased occurrence of intracellular enterovirus-RNA in peripheral blood lymphocytes (PBL) from type 1 diabetic children [5] and new evidence of enteroviral infection in pancreatic islets from deceased patients with type 1 diabetes [6] strongly suggests the possibility of the involvement of persistent enterovirus infection in the development of type 1 diabetes.

 Virus infections induce the production of type I interferons (IFN-*α*/*β*), which through the IFN receptor-mediated JAK-STAT signal transduction pathway [7] induce OAS and PKR genes encoding the key antiviral enzymes 2′5′-oligoadenylate synthetase (2′5′AS) and proteinkinase p68 (PKR). Both 2′5′AS and PKR are expressed as latent enzymes, and their enzymatic activities require a cofactor, double-stranded RNA (dsRNA), an intermediate usually generated by viral replication in virus infected cells. In the presence of dsRNA, the activated 2′5′AS polymerizes ATP into 2′-5′linked oligoadenylates (2′5′A), which in turn bind and activate a latent endoribonuclease, RNaseL. Activated RNaseL degrades cellular and viral RNA, thereby inhibiting protein synthesis [8]. Similarly, PKR, when activated, homodimerizes and autophosphorylates, leading to phosphorylation of eukaryotic initiation factor 2 (eIF2) and inhibition of cellular protein synthesis [9].

 Human 2′5′AS comprises a family of at least six enzyme isoforms, encoded by a cluster of three OAS genes, that is, OAS1-p42, p44, p46 and p48, OAS2-p69/p71, and OAS3-p100 [10]. Recently we have described a new splice variant, adding a new enzyme isoform to this family: OAS1-p52 [11]. We have suggested that processes within the antiviral 2′5′AS system might contribute to the etiopathogenesis of type 1 diabetes [12]. In several independent studies, we observed significantly increased 2′5′AS activity in patients with type 1 diabetes compared to healthy controls [12, 13]. More recently, we found a highly significant association between basal 2′5′AS activity and an A/G splice acceptor site single nucleotide polymorphism (SNP) in the *OAS1* gene, indicating that basal activity of this enzyme is strongly genetically controlled [11]. We have also reported that the frequencies of genotypes carrying the genetic variant associated with elevated activity were significantly higher in individuals with type 1 diabetes than in nondiabetic control subjects [14]. Thus, our original findings of persistently elevated 2′5′AS activity in type 1 diabetes patients compared to controls [12, 13] were at least partly genetically determined. In other words, elevated 2′5′AS activity might be due to both genetic and environmental factors. However, it is unclear how elevated 2′5′AS activity predisposes to the development of autoimmune type 1 diabetes.

 A number of studies have implicated interferon-*α* (IFN-*α*) as a potential mediator of type 1 diabetes pathogenesis. We have shown 2′5′AS activity to be highly induced by IFN-*α* and poly(I:C) (polyinosinic:polycytidylic acid, a synthetic dsRNA) in insulin-producing RIN rat beta cell lines [15]. Furthermore, we demonstrated that IFN-*α* sensitivity was not solely a characteristic of cultured cell lines, since it was also present in PBL from diabetes-prone BB rats [16] and in freshly isolated rat islets and rat beta cells [17]. In human islets, other investigators have shown that IFN-*α* was produced de novo by enterovirus-infected beta cells, but not by enterovirus-infected alpha cells [18]. From those data and ours, it can be concluded that beta cells not only are highly responsive to IFN-*α* and produce significant 2′5′AS activity, but also have the capacity to synthesize IFN-*α* [18]. It is therefore surprising that despite this highly reactive antiviral defense system, beta cells display an increased vulnerability to virus infection [18, 19]. Several studies have shown presence of IFN-*α* in human islets, localized to beta cells, from pancreases of deceased individuals both with acutely-developing and with long-standing type 1 diabetes [20, 21]. Since IFN-*α* has a very short half-life, the persistent presence of this cytokine in the islets could reflect chronic or repeated virus infections and result in continuous 2′5′AS gene activation. More recent studies have demonstrated the presence of enteroviruses in human beta cells of type 1 diabetic patients [6, 22, 23], which provides direct evidence that specific viral infection of beta cells plays an important role in the development of autoimmune diabetes. dsRNAs are produced during replication of both DNA and RNA viruses [24]. The viral mimic poly(I:C) is a synthetic dsRNA that can stimulate immune responses similar to those observed during virus infections. Recently it has been shown that the immune response induced by dsRNA or poly(I:C) is mediated by Toll-like receptors (TLRs), especially TLR3 [25]. It has also been shown that poly(I:C) can induce IFN-*α* production [26, 27], and that IFN-*α* induces interferon-stimulated genes (ISGs) including 2′5′AS and PKR [28–30], thus mediating innate immune responses.

Murine beta TC3 and alpha TC3 cell lines are transgenetically derived from insulinoma and glucagonoma tumor cells, and produce insulin and glucagon, respectively, in response to normal physiologic signals. In the present study, we used these two cell lines as a model to ask whether functional differences between pancreatic beta and alpha cells in their innate antiviral immune response could explain the beta cell target specificity characterizing the autoimmune destruction seen in type 1 diabetes. More specifically, we tested the hypothesis that pancreatic beta and alpha cells differ from each other in their 2′5′AS and PKR responses and their sensitivity to apoptosis when stimulated with IFN-*α* and/or poly(I:C).

## 2. Materials and Methods

### 2.1. Reagents

Poly(I:C), (*α* ^32^P)-ATP, and (*γ* ^32^P)-ATP were from AmershamPharmacia. Creatinekinase and creatine phosphate were purchased from Boehringer-Mannheim (Mannheim, Germany). DTT, Tris, FCS, penicillin, and streptomycin were from Invitrogen. Polygram Cel 300 PEI (polyetheleneimine) chromatography plates were from Machery-Nagel (Duren, Germany). Mouse interferon-*α* were from R&D Systems. BCA (bicinchoninic acid) and enhanced ECL kit were from Pierce.

### 2.2. Cell Culture and Stimulation of Cells with IFN-*α*

Murine beta TC3 and alpha TC3 cell lines were kindly provided by Dr. D. Hanahan, University of California, San Francisco. Beta TC3 cells were maintained in Dulbecco's modified Eagles' medium (DMEM) with 1,000 mg/L D-glucose, penicillin (100 U/mL), and streptomycin (100 *μ*g/mL) supplemented with 10% fetal bovine serum (FBS) at 37°C in 5% CO_2_ atmosphere. Alpha TC3 cells were cultured in DMEM medium containing 4,500 mg/L D-glucose and 15% FBS. At near confluency, medium containing the indicated amounts of IFN-*α* was added. After 2, 6, 12, and 24 hours of incubation in the presence or absence of 500 U/mL IFN-*α*, the cells were trypsinized and prepared for analysis of 2′5′AS activity.

### 2.3. Construction of Mouse IFN-*α*4 and TLR3 cDNAs-Containing Vectors pcDNA3.1-mIFN-*α*4 and pcDNA3.1-mTLR3

The whole length of murine IFN-*α*4 and TLR3 cDNAs were amplified by RT-PCR from total RNA extracted from beta TC3 cells treated with 500 U/mL IFN-*α*. The PCR primers were 5′-CTCGAGACCAGCATCTACAAGACCCA-3′ (forward, CTCGAG-XhoI) and 5′-GGATCCTCCACACTTTGTCTCAGGAC-3′ (reverse, GGATCC-BamHI) for mouse IFN-*α*4 (GenBank accession number X01973) and 5′-CTCGAGCACCCATAATCTGGGCTGAA-3′ (fwd, CTCGAG-XhoI) and 5′-GGATCCCCCTTTACCGACTCCAAATC-3′ (rev, GGATCC-BamHI) for mouse TLR3 (GenBank accession number NM_126166). The amplified cDNAs were inserted into the mammalian vectors, pcDNA3.1 (Invitrogen) using the restriction enzyme XhoI and Bam HI sites. The final mIFN-*α*4 and mTLR3 constructs were confirmed by DNA sequencing.

### 2.4. Transfection of Murine Pancreatic Beta TC3 and Alpha TC3 Cells

Transient transfection of the pcDNA3.1-mIFN-*α*4 and pcDNA3.1-mTLR3 vectors as well as poly(I:C) into murine pancreatic beta TC3 and alpha TC3 cells was performed using Lipofectamine 2000 (Invitrogen) according to manufacturer instructions. The mammalian expression vector pcDNA3.1 was used as a negative control. For transfection, 8 *μ*g of each of the plasmids and 5 *μ*g of poly(I:C) were added to 60 mm Petri dishes containing approximately 3 × 10^6^ cells in DMEM supplemented with 10% or 15% FBS. After 24 hours incubation, the transfected cells were trypsinized and prepared for analysis of 2′5′AS enzyme activity and Western blots. Transfection efficiency was monitored by including a beta-galactosidase-containing vector (pcDNA3.1-beta-gal, 0.6 *μ*g) during transfection of beta TC3 and alpha TC3 cells. The transfection efficiency was determined by assaying the cell lysates for beta-galactosidase activity (*A*_405_) and by normalizing to total protein (*A*_280_). Transfection efficiencies were similar in beta and alpha TC3 cells; there were no significant differences between alpha and beta cells.

### 2.5. Immunoblot Analysis

Cell proteins were separated by 12% SDS-PAGE and transferred to nitrocellulose membranes (Sigma). After blocking with 5% bovine serum albumin for 1 hour, the membranes were incubated with rabbit polyclonal antibody against recombinant human 2′5′AS protein isolated from infected insect cells using a Baculovirus expression system. This polyclonal antibody cross-reacts with murine 2′5′AS, detecting the murine p42 isoform, corresponding to a molecular weight of 42 kDa. For determination of PKR, a polyclonal antibody against human PKR was used. For detection of cell apoptosis after treatment with IFN-*α* and poly(I:C), a polyclonal antibody against mouse caspase 3 (Cell Signaling) was employed. Blots were incubated with horseradish-conjugated secondary antibodies followed by enhanced chemiluminescence detection (Pierce). The blots were scanned and quantitated by use of the Quantity One 1-D Image analysis software (BioRad). The % increase in protein expression was assessed by comparing the density of protein bands, normalized to the housekeeping gene GAPDH.

### 2.6. Assay of 2′5′AS and PKR Enzyme Activity

2′5′AS activity was determined in lysates of cultured beta TC3 and alpha TC3 cells as described previously [17]. Trypsinized cells were washed three times with PBS and lysates were prepared with 0.5% NP-40. Protein concentration of each cell lysate was determined with the BCA assay and adjusted to 1.5–3.0 mg/ml before measuring enzyme activity. For assay of 2′5′AS activity, lysates were incubated with 0.15 *μ*Ci *α* ^32^P-ATP for 120 minutes at 30°C in the presence of 100 *μ*g/mL poly(I:C). The generated 2′5′-oligoadenylates (2′5′A) were separated by ascending TLC on PEI plates in a Tris buffer (2 mM, pH 8.62). Enzyme activity was expressed in units per milligram protein. One unit of enzyme activity is defined as nanomoles ATP converted per minute. For assay of PKR activity, lysates were incubated with 0.15 *μ*Ci *α* ^32^P-ATP for 120 minutes at 30°C in the presence of 0.1 *μ*g/mL poly(I:C). Quantitation of PKR autophosphorylating activity was performed by ^32^P-ATP autoradiography.

### 2.7. Apoptosis Analysis by FACS

For FACS analysis of apoptosis, beta TC3 and alpha TC3 cells at a density of 3 × 10^5^/well (24 well plates) were transiently transfected with 0.8 *μ*g of pcDNA3.1-mIFN-*α*4 and 0.8 *μ*g of poly(I:C). The empty plasmid pcDNA3.1 was used as a negative control. After 24 hours incubation, the cell samples were analyzed with the Annexin-V-PE and 7-AAD staining kit (BD Biosciences) according to manufacturer instructions. Briefly, medium was removed from wells and cells were washed in PBS, trypsinized and spun down. Cell pellets were resuspended in 1 x binding buffer at a concentration of 1 × 10^6^/mL. 5 *μ*L of annexin-V-PE, and 7-AAD was added to 100 *μ*L of cell suspension. The mixture was incubated at room temperature in the dark for 15 minutes, washed once and FACS buffer was added. The samples were then analyzed in a FACSCalibur flow cytometer (BD Biosciences). Twenty thousand cells per sample were collected.

### 2.8. Statistical Analysis

All results are presented as mean ± SD of three independent experiments. Differences between groups were examined for statistical significance using one-way analysis of variance (ANOVA). Percentage increases in protein concentration or enzyme activity after 24 hours stimulation with IFN-*α* were calculated relative to non-stimulated values, with 100% indicating no increase. Values of *P* < .05 were considered to be significant.

## 3. Results

### 3.1. IFN-*α* Induced Higher 2′5′AS Protein Level and Enzyme Activity in Beta TC3 than in Alpha TC3 Cells

**Figure 1:**
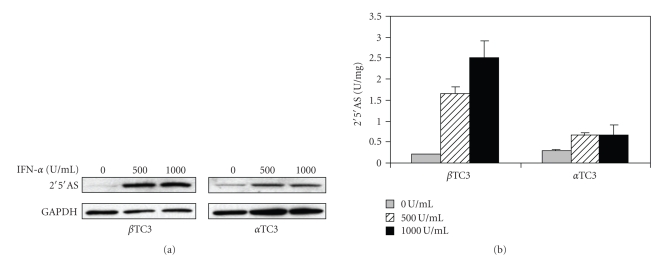
Protein expression and activity of 2′5′AS in non-stimulated and IFN-*α* stimulated beta TC3 and alpha TC3 cells. After 24 hours of IFN-*α* stimulation, cell lysates were prepared. (a) For immunoblot, cell extracts were separated on 12% SDS-PAGE and transferred to nitrocellulose membranes. The polyclonal antibody specific to human 2′5′AS (cross-reactive with murine 2′5′AS) was used to detect the expressed proteins. The polyclonal antibody against mouse GAPDH was used to quantitate the loading amounts; (b) For assay of 2′5′AS activity, lysates were incubated with 0.15 *μ*Ci *α* ^32^P-ATP for 120 minutes at 30°C in the presence of 100 *μ*g/ml poly(I:C), and then separated by ascending TLC. The generated 2′5′-oligoadenylates (2′5′A) were quantitated by PhosphorImager analysis. Enzyme activity was expressed in units per milligram protein (*n* = 3).

To determine whether IFN-*α* stimulates expression of 2′5′AS, immunoblot and enzyme assays were performed on lysates of beta and alpha TC3 cells after 24 hours of IFN-*α* treatment. As shown in Figure 1, expressed protein levels and enzyme activities of 2′5′AS were increased after IFN-*α* stimulation in both beta TC3 and alpha TC3 cells. However, the increased 2′5′AS protein levels (Figure 1(a)) and enzyme activities (Figure 1(b)) were significantly higher in beta than in alpha TC3 cells. In addition, the increase of 2′5′AS protein/activity in response to IFN-*α* stimulation was shown to be more dose-dependant in beta TC3 cells than in alpha TC3 cells.

### 3.2. 2′5′AS Activity Was Highly Induced by IFN-*α* in Beta TC3 Cells but Not in Alpha TC3 Cells

**Figure 2:**
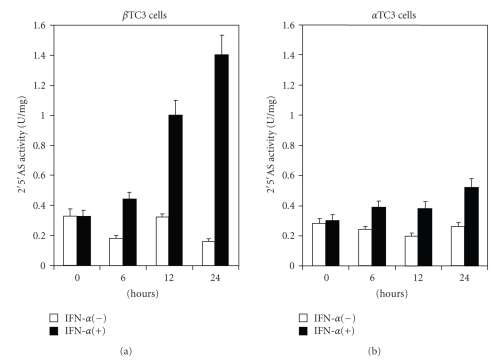
Enzyme activity of 2′5′AS in non-stimulated and IFN-*α* stimulated beta TC3 and alpha TC3 cells. Beta TC3 (a) and alpha TC3 (b) cells were incubated in the presence or absence of 500 U/ml of IFN-*α*. After the indicated time periods, cell lysates were prepared. The lysates were incubated with 0.15 *μ*Ci *α* ^32^P-ATP for 120 minutes at 30°C in the presence of 100 *μ*g/mL poly(I:C), and then separated by ascending TLC. The generated 2′5′A were quantitated by PhosphorImager analysis. Enzyme activity was expressed in units per milligram (*n* = 4, standard deviations varied between 6–13%).

To further investigate the increased expression of 2′5′AS following IFN-*α* induction, time-course studies were performed. Figure 2 shows the 2′5′AS enzyme activity in lysates of cultured beta TC3 (Figure 2(a)) and alpha TC3 cells (Figure 2(b)) in the presence or absence of 500 U/ml IFN-*α* for the indicated time periods. In beta TC3 cells, there was a strong time-dependent increase in 2′5′AS activity in response to IFN-*α* stimulation, ranging from 693% to 844% at 24 hours (*n* = 4) (SD varying between 6–13% in different cell cultures). In alpha TC3 cells, however, there was a very weak increase in activity, ranging from 178% to 209% at 24 hours (*n* = 4). The difference between beta and alpha TC3 cells in mean maximal 2′5′AS activity was statistically highly significant (769% compared to 189%, *P* < .001; see summary in Table 1). Constitutive (non-stimulated) 2′5′AS activity was similar in the two cell types (Figure 2).

### 3.3. PKR Protein Level and Enzyme Activity Were Upregulated Similarly in Beta TC3 and Alpha TC3 Cells

**Figure 3:**
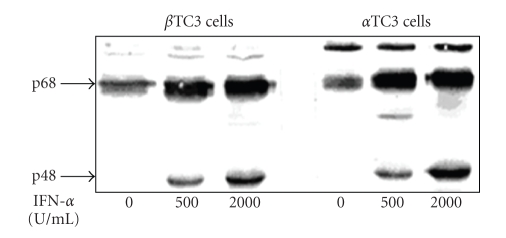
Protein expression of PKR in non-stimulated and IFN-*α* stimulated beta TC3 and alpha TC3 cells. After beta TC3 and alpha TC3 cells were stimulated with 500 U/mL (lane 2 and 5) and 2000 U/mL (lane 3 and 6) of IFN-*α* for 24 hours, prepared lysates were subjected to 12% SDS-PAGE, transferred onto nitrocellulose, and immunostained with a polyclonal antibody specific for human PKR. Two protein bands were detected; the p48 subunit is the degradation product of PKR (p68). The relative levels of protein were determined by quantifying the immunoblots using the Quantity One 1-D Image software (see Table 1).

**Figure 4:**
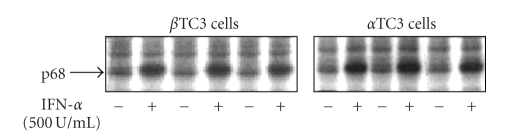
Enzyme activity of PKR in non-stimulated and IFN-*α* stimulated beta TC3 and alpha TC3 cells. Beta TC3 and alpha TC3 cells were incubated with or without addition of 500 U/mL of IFN-*α* into the medium for 24 hours in three independent experiments. The lysates were subjected to an autophosphorylation assay with *γ* ^32^P-ATP. Incubation time was 1 hour. Proteins were fractionated by 12% SDS-PAGE and radioactive bands were visualized by autoradiography. After the gels were dried, the protein bands were cut and the radioactivity was quantitated in a scintillation counter.

PKR is also an IFN-*α* stimulated gene, therefore similar experiments were performed to determine PKR expression in response to IFN-*α* treatment. The expression level of the PKR protein was determined as the sum of the p68 PKR molecule and the p48 immune reactive degradation product of the PKR molecule, as previously described [31, 32] (Figure 3). Because PKR activity is inhibited at higher poly(I:C) concentrations, a low concentration of 0.1 *μ*g/ml was used in all incubations for the measurement of PKR activity. Figure 3 shows the upregulation of PKR protein in beta TC3 and alpha TC3 cells in the presence of 500 or 2000 U/ml IFN-*α*. PKR protein levels were upregulated by 166% and 192%, respectively, in beta TC3 cells and by 171% and 208% in alpha TC3 cells. Thus, similar amounts of PKR protein were induced by IFN-*α* stimulation of both cell types. Furthermore, the increase in autophosphorylating activity of PKR after stimulation with 500 U/ml IFN-*α* showed no statistically significant difference between beta TC3 cells (151% ± 13, *n* = 3) and alpha TC3 cells (121% ± 19, *n* = 3) (Figure 4 and Table 1).

### 3.4. Poly(I:C) Induced Expression of 2′5′AS in Beta TC3 but Not in Alpha TC3 Cells

**Figure 5:**
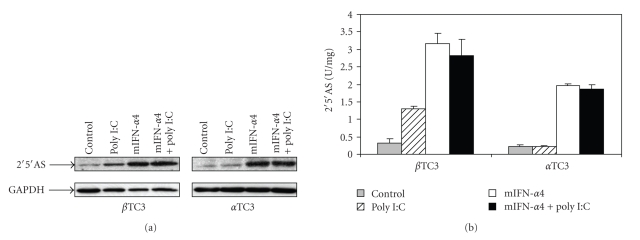
Protein expression and activity of 2′5′AS in beta TC3 and alpha TC3 cells transfected with mouse IFN-*α*4-containing vector and poly(I:C). Beta TC3 and alpha TC3 cells were transiently transfected with pcDNA3.1-mIFN-*α*4, poly(I:C), and pcDNA3.1-mIFN-*α*4 plus poly(I:C), respectively. pcDNA3.1 empty vector was used as a negative control. After 24 hours, cell lysates were prepared. Immunoblot (a) and 2′5′AS activity assay (b) were performed as described under the materials and methods.

Since poly(I:C) is known to be a potent IFN-*α* inducer [26, 27], poly(I:C) was transiently transfected into beta TC3 and alpha TC3 cells, without or with mouse IFN-*α*4 cDNA-containing plasmid, to determine the effect on expression of 2′5′AS protein. Murine IFN-*α*4 is an immediate-early interferon and can be produced by naïve cells upon viral infection. In order to induce higher activity of 2′5′AS, we used an IFN-*α*4-containing plasmid overexpressing type I IFN in the cell lines to simulate the situation of cells experiencing acute viral infection. As shown in Figure 5, introduction of poly(I:C) alone produced a marked increase in 2′5′AS protein levels (Figure 5(a)) and enzyme activity (Figure 5(b)) in beta TC3 cells, but not in alpha TC3 cells. Although transfection of mouse IFN-*α*4 cDNA resulted in a significant increase of 2′5′AS expression in both beta and alpha TC3 cells, the magnitude of the increase was much higher in beta than in alpha TC3 cells (Figure 5). However, co-transfection of poly(I:C) and pcDNA3.1-mIFN-*α*4 did not result in any further increase of 2′5′AS protein (Figure 5(a)) or enzyme activity (Figure 5(b)) when compared with mIFN-*α*4 treatment alone in either beta TC3 or alpha TC3 cells.

### 3.5. Induction of 2′5′AS by Poly(I:C) in Beta TC3 Cells Was Not Augmented by TLR3 Overexpression

**Figure 6:**
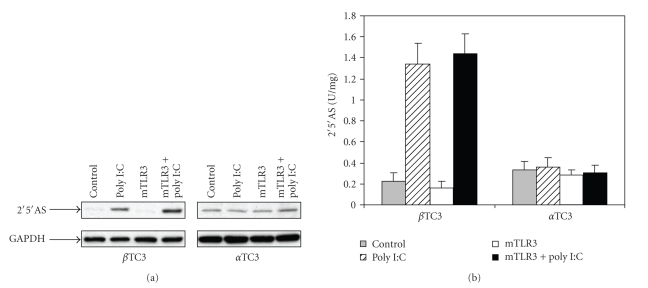
Protein expression and activity of 2′5′AS in beta TC3 and alpha TC3 cells transfected with mouse TLR3-containing vector and poly(I:C). Beta TC3 and alpha TC3 cells were transiently transfected with pcDNA3.1-mTLR3, poly(I:C), and pcDNA3.1-mTLR3 plus poly(I:C), respectively. pcDNA3.1 empty vector was used as a negative control. After 24 hours, cell lysates were prepared. Immunoblot (a) and 2′5′AS activity assay (b) were performed as described in the Materials and Methods section.

To investigate whether TLR3 plays a role in the upregulation of 2′5′AS by poly(I:C), similar transfection experiments were performed in beta TC3 and alpha TC3 cells with mouse *TLR3* cDNA-containing vector. Results presented in Figure 6 confirmed that poly(I:C) alone significantly increases 2′5′AS protein level and enzyme activity in beta TC3 cells, but not in alpha TC3 cells. In beta TC3 cells, co-transfection of poly(I:C) and pcDNA3.1-mTLR3 appeared to induce higher expression of 2′5′AS (Figure 6(a)), but no statistically significant difference was observed between 2′5′AS activity in the poly(I:C)-treated only and the co-transfection groups (Figure 6(b)). In alpha TC3 cells, co-transfection of poly(I:C) and mTLR3 cDNA had no effect on expression of 2′5′AS (Figure 6). Basal expression of TLR3 mRNA as determined by real-time RT-PCR was similar in the two cell lines (data not shown).

### 3.6. IFN-*α* and Poly(I:C) Induced Apoptosis in Beta TC3 Cells but Not in Alpha TC3 Cells

**Figure 7:**
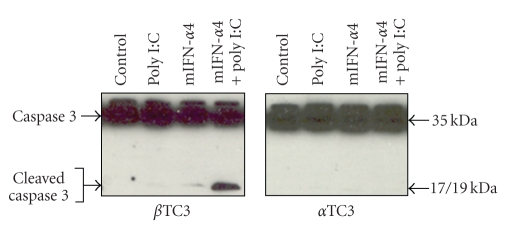
Induction of apoptosis in beta TC3 and alpha TC3 cells after transfection of IFN-*α*4-containing vector and poly(I:C). After beta TC3 and alpha TC3 cells were transiently transfected with pcDNA3.1-mIFN-*α*4, poly(I:C), and pcDNA3.1-mIFN-*α*4 plus poly(I:C) for 24 hours, respectively, induction of apoptosis was detected by immunoblots. pcDNA3.1 empty vector was used as a negative control. Cells lysates were separated on 15% SDS-PAGE and transferred to nitrocellulose membranes. The polyclonal antibody specific to mouse caspase 3 was used to detect both the intact as well as the cleaved caspase 3 proteins.

**Figure 8:**
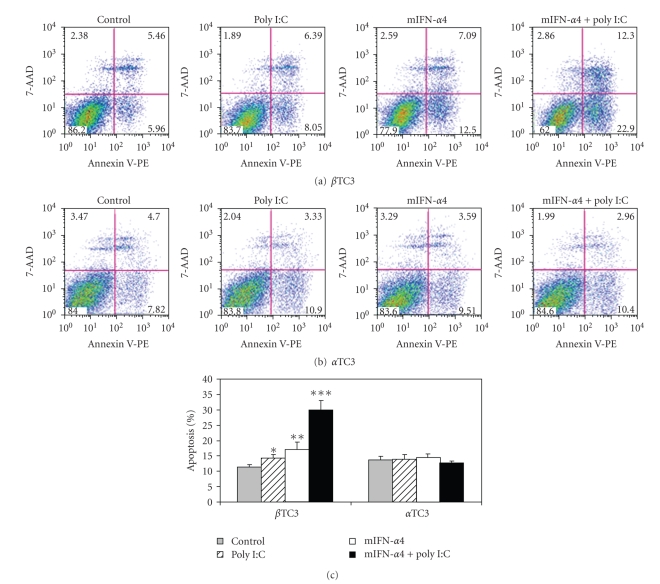
FACS analysis of apoptosis induction in beta TC3 and alpha TC3 cells after transfection of mouse IFN-*α*4-containing vector and poly(I:C). Beta TC3 and alpha TC3 cells were transiently transfected with pcDNA3.1-mIFN-*α*4, poly(I:C), and pcDNA3.1-mIFN-*α*4 plus poly(I:C), respectively. pcDNA3.1 empty vector was used as a negative control. After 24 hours, the cell samples were stained with the Annexin-V-PE and 7-AAD kit, and induction of apoptosis was assayed by FACS analysis. (a) Beta TC3 cells; (b) alpha TC3 cells; (c) the percentage of apoptosis of induction in beta TC3 and alpha TC3 cells after transfection. *P < .05, **P < .005, ***P < .0001 (transfected group versus control, *n* = 3).

In order to determine whether high expression of 2′5′AS induces apoptosis, immunoblot and flow cytometry were used to demonstrate the presence of apoptosis in transfected cells. Cleaved caspase 3 was detected by specific anti-caspase 3 antibody following transfection of poly(I:C), mIFN-*α*4, or both poly(I:C) plus mIFN-*α*4 into beta TC3 and alpha TC3 cells. As shown in Figure 7, co-transfection of pcDNA3.1-mIFN-*α*4 and poly(I:C) induced marked apoptosis, but only in beta TC3 cells. Apoptosis induction as measured by FACS analysis produced similar results (Figure 8) to those using immunoblot analysis. Alpha TC3 cells were resistant to apoptosis; neither stimulation with poly(I:C), mIFN-*α*4 nor co-stimulation with poly(I:C) and mIFN-*α*4 induced detectable apoptosis as compared with control cells (Figures 8(b) and 8(c)). However, beta TC3 cells showed significant susceptibility to apoptosis (Figures 8(a) and 8(c)). In these cells, stimulation with poly(I:C) induced apoptosis in 2.90% more of the treated cells than the untreated control cells (*P* < .05), mIFN-*α*4 induced 5.87% more cellular apoptosis (versus controls, *P* < .005), and co-transfection of both mIFN-*α*4 and poly(I:C) induced 18.76% apoptosis (versus controls, *P* < .0001).

## 4. Discussion

In the present study, we showed that there is a functional difference between mouse pancreatic beta and alpha TC3 cells in 2′5′AS induction in response to IFN-*α* stimulation. Firstly, elevations of 2′5′AS protein level and enzyme activity were significantly higher in beta than in alpha TC3 cells following IFN-*α* stimulation (Figure 1). Furthermore, only beta TC3 cells showed strongly dose- and time-dependant increases in 2′5′AS enzyme activity after IFN-*α* treatment (Figures 1(b) and 2). Secondly, introduction of poly(I:C) (a synthetic dsRNA) stimulated 2′5′AS expression and activity in beta TC3 cells but not in alpha TC3 cells (Figures 5 and 6). Finally, overexpression of IFN-*α*, with or without poly(I:C), induces apoptosis in beta TC3 cells but not in alpha TC3 cells (Figures 7 and 8). It is unlikely that the observed differences between pancreatic beta and alpha cells are due to use of transformed cell lines, since previously we have observed increased 2′5′AS activity after IFN-*α* stimulation in *primary* rat beta cells but not in rat non-beta (mainly alpha) islet cells [17]. The results suggest that pancreatic beta cells have much more intrinsically responsive elements in activation of the 2′5′AS system than alpha cells.

 However, no difference between beta and alpha TC3 cells was observed for induction of PKR, another dsRNA and IFN-*α*-dependent antiviral enzyme. The expressed protein level and enzyme activity of PKR were similarly upregulated in beta and alpha TC3 cells. In both cell types, p68 and its p48 degradation product were detected in accordance with earlier findings [31, 32]. Expression of PKR has been shown in mouse, rat, and human islets after dsRNA stimulation and coxsackievirus infection [33, 34]. Although both 2′5′AS and PKR are dependent on dsRNA as a cofactor for activation, it is well known that they differ significantly in the dsRNA concentration needed for activation [9]. PKR is activated at dsRNA concentrations about 100 times lower than those needed for 2′5′AS activation, and becomes inhibited at higher dsRNA concentrations [35]. Thus, at low cellular dsRNA concentrations PKR-dependent inhibition of protein synthesis may increase the risk of apoptosis, but at higher concentrations the 2′5′AS-RNaseL system [36, 37] may promote apoptotic processes*. *Importantly, we analyzed PKR activity at very low dsRNA concentrations rather than at higher inhibitory concentrations, and found similar PKR activity in beta TC3 and alpha TC3 cells (Figure 4 and Table 1). This result suggests that if there is a difference in antiviral activity-mediated apoptosis between pancreatic beta and alpha cells, the differences are not due to PKR-induced apoptosis.

 Although expression and activity of 2′5′AS were similar after transfection of IFN-*α* vector alone compared to co-transfection of IFN-*α* plus poly(I:C), both in alpha and beta TC3 cells (Figure 5), we noted that in beta TC3 cells, caspase 3 cleavage and apoptosis were very significantly elevated after IFN-*α plus* poly(I:C) co-transfection as compared to IFN-*α* alone (Figures 7 and 8). At the moment, we do not know the reason for this discrepancy with respect to 2′5′AS activity and apoptosis-induction in the co-transfected beta TC3 cells. The 2′5′AS enzyme activity in beta cells appeared to reach maximum biological levels with IFN-*α* alone and be nonresponsive to additional stimulus with poly(I:C), while the apoptotic response of IFN-*α*-stimulated beta cells was further augmented by poly(I:C). This could be due to toxicity of poly(I:C) itself on beta (but not alpha) cells, although poly(I:C) alone had only a limited effect on beta cell apoptosis (Figure 8(c)). Alternatively, this could be due to some other unidentified pro-apoptotic effect induced by dsRNA structures, but independent of 2′5′AS enzyme activity. Interestingly, in humans it has been reported that the p48 isoform of 2′5′AS (encoded by the *OAS1* gene) has pro-apoptotic activity independent of its synthetase activity [38], and that a 2′5′AS-like protein (encoded by the closely-linked *OASL* gene) responds to virus infection but has no synthetase activity [39, 40]. However, the above-noted discrepancy in the co-transfection experiments does not invalidate the suggestion that the higher IFN-*α*-stimulated 2′5′AS response of beta cells compared to alpha cells (Figures 2 and 5) contributes to the greater apoptosis observed following IFN-*α* transfection in beta cells compared to alpha cells (Figure 8). The situation is complex, since poly(I:C) is both a cofactor for 2′5′AS activation and a potent IFN-*α* inducer. As shown in Figure 5, 2′5′AS activity in *alpha* cells was not affected by poly(I:C) alone, suggesting that poly(I:C) could not induce IFN-*α* production in alpha cells. This assumption was consistent with the observation that IFN-*α* could be detected in enterovirus-infected beta cells but not in enterovirus-infected alpha cells [18]. In humans, IFN*α* induces more than 40 known IFN-stimulated genes (ISG), among which dsRNA-dependent antiviral enzymes 2′5′AS and PKR have been associated with apoptotic cell death. Since there were no differences in PKR protein level and enzyme activity between beta and alpha cells, we propose that the beta cell's stronger 2′5′AS response to poly(I:C) alone and IFN-*α* alone (Figures 2 and 5) is a critical contributor to the greater apoptosis seen in beta compared to alpha pancreatic cells.

**Figure 9:**
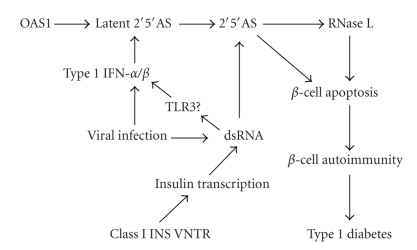
Schematic presentation of a model of induction of beta cell apoptosis during viral infections. Virus infections result in production of IFN-*α* and dsRNA. IFN-*α* induces expression of 2′5′AS though the classical IFN-*α* signal transduction pathway, and dsRNA can lead to induction of IFN-*α*/*β* and to expression of 2′5′AS via TLR3-mediated signaling pathway. In the presence of dsRNA, 2′5′AS is activated. The 2′5′A oligomers generated by 2′5′AS stimulate RNaseL-induced apoptosis, which results in subsequent beta cell autoimmunity. 2′5′AS can also promote cellular apoptosis via an enzyme activity-independent pathway. In genetically predisposed individuals, increased insulin gene expression may produce dsRNA-like molecules that contribute to enhancement of the activation and induction of 2′5′AS in beta cells.

What are the intrinsic factors in beta cells that contribute to higher activation of the 2′5′AS system in response to IFN-*α* and poly(I:C)? One of the possible contributors is a characteristic feature of the pancreatic beta cell, that is, its high rate of insulin synthesis. It is known that during virus infection, insulin synthesis increases as part of the stress response. In addition, during development of type 1 diabetes, insulin synthesis increases in an attempt to reduce high blood glucose levels created by a diminishing beta cell mass. We have preliminary evidence (described in what follows) that increased insulin synthesis augments the effect of IFN-*α* or poly(I:C) on beta cell 2′5′AS activity and apoptosis, possibly through the generation of dsRNA structures during insulin gene transcription. As mentioned earlier, 2′5′AS is expressed as a latent enzyme that requires dsRNA (e.g., produced by replicating virus) to become activated. Binding of dsRNA to 2′5′AS generates a conformational change in 2′5′AS that is necessary for enzyme activation [41]. There is now data supporting the existence of *endogenous* dsRNA [42]. Furthermore, it has been show that small single-stranded RNA ligands can also activate 2′5′AS [43]. Single-stranded mRNA could also play a role in 2′5′AS activation, since mRNA has been reported to be a potent endogenous activator of Toll-like receptor 3 (TLR3), likely through secondary dsRNA-like structures [44]. We have preliminary data showing that beta TC3 cell apoptosis was significantly increased when beta cells were cultured in high glucose-containing medium (20 mmol/L) compared with low glucose medium (5 mmol/L), followed by treatment with either IFN-*α* (1000 U/mL) or poly(I:C) (50 *μ*g/mL) for 24 hours (Bonnevie-Nielsen et al., unpublished data), suggesting that the higher insulin transcription rate stimulated by high glucose significantly augmented beta cell sensitivity to apoptosis. Alpha TC3 cells showed little apoptotic response to any treatment or glucose condition (note that these results also implied that high glucose *per se* does not promote apoptosis). Other preliminary studies showed that beta cells treated with poly(I:C) for 24 hours displayed significantly higher 2′5′AS activity (approximately 2.5 times higher) when cultured in high glucose compared to low glucose medium (Bonnevie-Nielsen et al., unpublished data). Taken together, these findings suggested that a higher insulin transcription rate had a strong effect on activation of 2′5′AS by poly(I:C) and on beta sensitivity to apoptosis when treated with poly(I:C) or IFN-*α*. Thus, we hypothesize that increased insulin gene transcription in beta cells during virus infection and/or during development of type 1 diabetes could generate dsRNA-like structures that augment 2′5′AS activity and contribute to beta cell apoptosis. Consistent with this hypothesis, it is well established that a genetic polymorphism (variable number tandem repeat, VNTR) in the promoter region of the human insulin gene alters susceptibility to type 1 diabetes, that class I (short-repeat) VNTR alleles are strongly associated with increased risk of developing type 1 diabetes [45, 46], and that class I alleles also are associated with an increased insulin gene transcription rate in pancreatic beta cells [47]. Thus, a higher rate of insulin transcription in persons with class I homozygous genotypes may genetically predispose them to diabetes by providing an increased endogenous source of dsRNA-like molecules that contribute to 2′5′AS activation in beta cells, particularly during virus infection (Figure 9). Clearly, more studies are needed to test the “high insulin transcription rate hypothesis” of beta cell sensitivity to apoptosis.

 During viral infection, IFN-*α* is induced and dsRNAs are generated. IFN-*α* stimulates antiviral activity of the innate immune system through activation of transcription of ISGs including PKR and 2′5′AS [21, 27, 29, 48]. dsRNA is not only an essential cofactor for activation of the antiviral enzymes, but also leads to induction of the antiviral enzymes either directly or via TLR3-mediated activation of IFN regulatory factor 3 (IRF-3) and NF-kB signaling pathways [49]. Although we found that TLR3 did not augment 2′5′AS induction by poly(I:C) in beta TC3 cells (Figure 6), others have reported that activation of TLR3 by dsRNA increased beta cell apoptosis via NF-kB and STAT-1 [50]. Thus, dsRNA may contribute to beta cell apoptosis through multiple pathways. Increased activity of the 2′5′AS-RNaseL system has been shown to induce apoptosis in several cell types and is an important mechanism through which virus replication and spreading is restricted [36, 37]. In addition, as mentioned earlier, one 2′5′AS isoform can promote cellular apoptosis independent of synthetase activation of RNaseL [38]. Although epidemiological and pathological studies have suggested that enteroviruses are associated with development of type 1 diabetes, the selective tropism of these viruses for pancreatic beta cells and the factors that determine the sensitivity of beta cells to virus infection are still not well understood. It has been reported that administration of poly(I:C) accelerated the development of diabetes in DP-BB rats, and there was a dose-relationship of poly(I:C) with serum IFN-*α* levels and the time of onset of diabetes [51]. IFN-*α* has been detected in residual pancreatic islets from deceased patients with recent-onset or long-term type 1 diabetes [20, 21] and IFN-*α* was reported to be a potential mediator of type 1 diabetes induced by poly(I:C) in C57BL/6 mice expressing the B7.1 costimulatory protein in islets [52]. Somewhat paradoxically, it has also been reported that induction of IFNs in virus-infected beta cells was critical for their survival during viral infection [18, 53]. Increased numbers of enteroviral infections have been reported in prediabetic individuals [2, 4, 54], and recently enteroviruses have been detected in pancreatic tissue from type 1 diabetic patients [6, 22, 23]. Taken together, these findings suggest that pancreatic viral infection may occur before and during onset of clinical diabetes, and that alterations of antiviral defense in virus-infected pancreatic beta cells may influence susceptibility to developing type I diabetes.

 Based on our data and a variety of clinical and epidemiological observations mentioned earlier, we therefore suggest that during infection with diabetogenic viruses, the 2′5′AS-RNaseL system in beta cells becomes activated by IFN-*α*, while production of dsRNA during virus replication as well as dsRNA-like molecules during increased insulin transcription lead to further induction of OAS genes (either directly or via the TLR3-mediated signaling pathway) and to activation of 2′5′AS by dsRNA (Figure 9). The markedly increased activity of the 2′5′AS-RNaseL system would be detrimental to beta cells, making them more prone to damage/death during viral infection. Subsequent minor-but-critical apoptosis of beta cells due to high 2′5′AS activity would allow sequestered beta cell antigens to be exposed to the immune system, which (in individuals who are genetically predisposed through, for example, HLA genes) could trigger an autoimmune response and ultimately destruction of the pancreatic beta cell mass. Genetic variants that elevate the constitutive 2′5′AS activity would also facilitate this process, such as the *OAS1* splice site polymorphism that we and others have reported to be associated with type 1 diabetes [14, 55], and the insulin VNTR associated with insulin gene transcription and with type 1 diabetes [45–47]. By contrast, the relatively low activity of the 2′5′AS-RNaseL system in alpha cells would not lead to any significant cell apoptosis during virus infections. Thus, we speculate that virus infections cause over-reactivity of the beta cell innate immune system, which facilitates beta cell apoptosis and autoimmunity. Measures aimed at regulating the beta cell antiviral response during the prediabetic phase in genetically high risk children could help preserve beta cell mass and thereby prevent or reduce the risk of developing type 1 diabetes.

## 5. Conclusions

In conclusion, this study showed that following IFN-*α* stimulation, increases in 2′5′AS protein level and enzyme activity were significantly higher in beta than in alpha TC3 cells (*P* < .001). It also demonstrated that poly(I:C) stimulated 2′5′AS enzyme activity in beta TC3 cells but not in alpha TC3 cells, and that co-transfection of IFN-*α* and poly(I:C) induced apoptosis in beta TC3 but not in alpha TC3 cells. The results suggest that the increased 2′5′AS activity of pancreatic beta cells could render them particularly vulnerable to apoptosis during virus infection and to subsequent autoimmune destruction.

## Figures and Tables

**Table 1:** Summary of mean percentage increases of protein levels and enzyme activities of 2′5′AS and PKR in beta TC3 and alpha TC3 cells after 24 hours 500 U/ml of IFN-*α* stimulation.

	2′5′AS (% increase ± SD)*		PKR (% increase ± SD)*	
	Beta TC3 cells (*n* = 4)	Alpha TC3 cells (*n* = 4)	Beta TC3 cells (*n* = 3)	Alpha TC3 cells (*n* = 3)
Protein	680 ± 70	214 ± 19	166 ± 10	171 ± 12
Activity	769 ± 78	189 ± 15	151 ± 13	121 ± 19

*100% indicates no change.

## Abbreviations

2′5′AS: 2′5′-oligoadenylate synthetase
dsRNA: Double-stranded RNA
IFN: Interferon
ISG: Interferon-stimulated gene
PKR: Proteinkinase p68
poly(I:C): Polyinosinic:polycytidylic acid
PBL: Peripheral blood lymphocytes
TLR3: Toll-like receptor 3.

## References

[1] Hyöty H, Hiltunen M, Knip M (1995). The childhood diabetes in Finland (DiMe) study group: a prospective study of the role of coxsackie B and other enterovirus infections in the pathogenesis of IDDM. *Diabetes*.

[2] Hiltunen MH, Hyöty M, Knip J (1997). Islet cell antibody seroconversion in children is temporally associated with enterovirus infections. *The Journal of Infectious Diseases*.

[3] Roivainen M, Knip M, Hyöty H (1998). Several different enterovirus serotypes can be associated with prediabetic autoimmune episodes and onset of overt IDDM: childhood diabetes in Finland (DiMe) study. *Journal of Medical Virology*.

[4] Lönnrot M, Korpela K, Knip M (2000). Enterovirus infection as a risk factor for *β*-cell autoimmunity in a prospectively observed birth cohort: the Finnish diabetes prediction and prevention study. *Diabetes*.

[5] Yin H, Berg A-K, Tuvemo T, Frisk G (2002). Enterovirus RNA is found in peripheral blood mononuclear cells in a majority of type 1 diabetic children at onset. *Diabetes*.

[6] Richardson SJ, Willcox A, Bone AJ, Foulis AK, Morgan NG (2009). The prevalence of enteroviral capsid protein vp1 immunostaining in pancreatic islets in human type 1 diabetes. *Diabetologia*.

[7] Schindler C, Darnell JE (1995). Transcriptional responses to polypeptide ligands: the JAK-STAT pathway. *Annual Review of Biochemistry*.

[8] Stark GR, Kerr IM, William BR, Silverman RH, Schreiber RD (1998). How cells response to interferons. *Annual Review of Biochemistry*.

[9] Samuel CE, Kuhen KL, George CX, Ortega LG, Rende-Fournier R, Tanaka H (1997). The PKR protein kinase—an interferon-inducible regulator of cell growth and differentiation. *International Journal of Hematology*.

[10] Justesen J, Hartmann R, Kjeldgaard NO (2000). Gene structure and function of the 2′-5′-oligoadenylate synthetase family. *Cellular and Molecular Life Sciences*.

[11] Bonnevie-Nielsen V, Field LL, Lu S (2005). Variation in antiviral 2′, 5′-oligoadenylate synthetase (2′5′AS) enzyme activity is controlled by a single-nucleotide polymorphism at a splice-acceptor site in the *OAS1* gene. *American Journal of Human Genetics*.

[12] Bonnevie-Nielsen V, Larsen ML, Frifelt JJ, Michelsen B, Lernmark Å (1989). Association of IDDM and attenuated response of 2′, 5′-oligoadenylate synthetase to yellow fever vaccine. *Diabetes*.

[13] Bonnevie-Nielsen V, Martensen PM, Justesen J (2000). The antiviral 2′, 5′-oligoadenylate synthetase is persistently activated in type 1 diabetes. *Clinical Immunology*.

[14] Field LL, Bonnevie-Nielsen V, Pociot F, Lu S, Nielsen TB, Beck-Nielsen H (2005). *OAS1* splice site polymorphism controlling antiviral enzyme activity influences susceptibility to type 1 diabetes. *Diabetes*.

[15] Bonnevie-Nielsen V, Gerdes A-M, Fleckner J, Petersen JS, Michelsen B, Dyrberg T (1991). Interferon stimulates the expression of 2′, 5′-oligoadenylate synthetase and MHC class I antigens in insulin-producing cells. *Journal of Interferon Research*.

[16] Bonnevie-Nielsen V, Husum G, Markholst H, Dyrberg T (1992). Double-stranded RNA, but not *α*-IFN accelerates onset of diabetes and restores 2′, 5′-oligoadenylate synthetase activity in the BB rat. *Pediatric and Adolescent Endocrinology*.

[17] Bonnevie-Nielsen V, Buschard K, Dyrberg T (1996). Differential responsiveness to interferon-*α*
in *β*-cells and non-*β*-cells. *Diabetes*.

[18] Chehadeh W, Kerr-Conte J, Pattou F (2000). Persistent infection of human pancreatic islets by coxsackievirus B is associated with alpha interferon synthesis in *β*-cells. *Journal of Virology*.

[19] Roivainen M, Rasilainen S, Ylipaasto P (2000). Mechanisms of coxsackievirus-induced damage to human pancreatic *β*-cells. *The Journal of Clinical Endocrinology & Metabolism*.

[20] Foulis AK, Farquharson MA, Meager A (1987). Immunoreactive *α*-interferon in insulin-secreting *β* cells in type 1 diabetes mellitus. *The Lancet*.

[21] Huang X, Yuan J, Goddard A (1995). Interferon expression in the pancreases of patients with type I diabetes. *Diabetes*.

[22] Ylipaasto P, Klingel K, Lindberg AM (2004). Enterovirus infection in human pancreatic islet cells, islet tropism in vivo and receptor involvement in cultured islet beta cells. *Diabetologia*.

[23] Dotta F, Censini S, van Halteren AGS (2007). Coxsackie B4 virus infection of *β* cells and natural killer cell insulitis in recent-onset type 1 diabetic patients. *Proceedings of the National Academy of Sciences of the United States of America*.

[24] Guidotti LG, Chisari FV (2001). Noncytolytic control of viral infections by the innate and adaptive immune response. *Annual Review of Immunology*.

[25] Medzhitov RC, Janeway C (2000). Innate immune recognition: mechanisms and pathways. *Immunological Reviews*.

[26] Verdijk RM, Mutis T, Esendam B (1999). Polyriboinosinic polyribocytidylic acid (poly(I:C)) induces stable maturation of functionally active human dendritic cells. *The Journal of Immunology*.

[27] van Pesch V, Lanaya H, Renauld J-C, Michiels T (2004). Characterization of the murine alpha interferon gene family. *Journal of Virology*.

[28] Rebouillat D, Hovanessian AG (1999). The human 2′, 5′-oligoadenylate synthetase family: interferon-induced proteins with unique enzymatic properties. *Journal of Interferon and Cytokine Research*.

[29] Levy DE, García-Sastre A (2001). The virus battles: IFN induction of the antiviral state and mechanisms of viral evasion. *Cytokine and Growth Factor Reviews*.

[30] Geiss G, Jin G, Guo J, Bumgarner R, Katze MG, Sen GC (2001). A comprehensive view of regulation of gene expression by double-stranded RNA-mediated cell signaling. *The Journal of Biological Chemistry*.

[31] Kadereit S, Galabru J, Robert N, Meurs EF, Hovanessian AG (1994). Characterization of an interferon-induced 48-kD protein immunologically related to the double-stranded RNA-activated protein kinase PKR. *Journal of Interferon Research*.

[32] Yang Y-L, Reis LFL, Paylovic J (1995). Deficient signaling in mice devoid of double-stranded RNA-dependent protein kinase. *EMBO Journal*.

[33] Blair LA, Heitmeier MR, Scarim AL, Maggi Jr. LB, Corbett JA (2001). Double-stranded RNA-dependent protein kinase is not required for double-stranded RNA-induced nitric oxide synthase expression or nuclear factor-*κ*B activation by islets. *Diabetes*.

[34] Flodstrom-Tullberg M, Hultcrantz M, Stotland A (2005). RNase L and double-stranded RNA-dependent protein kinase exert complementary roles in islet cell defense during coxsackivirus infection. *The Journal of Immunology*.

[35] Clemens MJ, Laing KG, Jeffrey IW (1994). Regulation of the interferon-inducible eIF-2 *α* protein kinase by small RNA's. *Biochimie*.

[36] Zhou A, Paranjape JM, Hassel BA (1998). Impact of RNaseL overexpression on viral and cellular growth and death. *Journal of Interferon & Cytokine Research*.

[37] Castelli JC, Hassel BA, Maran A (1998). The role of 2′5′-oligoadenylate-activated ribonuclease L in apoptosis. *Cell Death and Differentiation*.

[38] Ghosh A, Sarkar SN, Rowe TM, Sen GC (2001). A specific isozyme of 2′5′-oligoadenylate synthetase is a dual function proapoptotic protein of the Bcl-2 family. *The Journal of Biological Chemistry*.

[39] Marques J, Anwar J, Eskildsen-Larsen S (2008). The p59 oligoadenylate synthetase-like protein possesses antiviral activity that requires the C-terminal ubiquitin-like domain. *Journal of General Virology*.

[40] Melchjorsen J, Kristiansen H, Christiansen R (2009). Differential regulation of the OASL and OAS1 genes in response to viral infections. *Journal of Interferon and Cytokine Research*.

[41] Hartmann R, Justesen J, Sarkar SN, Sen GC, Yee VC (2003). Crystal structure of the 2′-specific and double-stranded RNA-activated interferon-induced antiviral protein 2′5′-oligoadenylate synthetase. *Molecular Cell*.

[42] Lehner B, Williams G, Campbell RD, Sanderson CM (2002). Antisense transcripts in the human genome. *Trends in Genetics*.

[43] Hartmann R, Norby PL, Martensen PM (1998). Activation of 2′-5′
oligoadenylate synthetase by single-stranded and double-stranded RNA aptamers. *Journal of Biological Chemistry*.

[44] Karikó K, Ni H, Capodici J, Lamphier M, Weissman D (2004). mRNA is an endogenous ligand for toll-like receptor 3. *The Journal of Biological Chemistry*.

[45] Bennett ST, Wilson AJ, Cucca F (1996). IDDM2-VNTR-encoded susceptibility to type 1 diabetes: dominant protection and parental transmission of alleles of the insulin gene-linked minisatellite locus. *The Journal of Autoimmunity*.

[46] Bennett ST, Wilson AJ, Esposito L (1997). Insulin VNTR allele-specific effect in type 1 diabetes depends on identity of untransmitted paternal allele. *Nature Genetics*.

[47] Vafiadis P, Bennett ST, Colle E, Grabs R, Goodyer CG, Polychronakos C (1996). Imprinted and genotype-specific expression of genes at the IDDM2 locus in pancreas and leucocytes. *Journal of Autoimmunity*.

[48] Müller U, Steinhoff U, Reis LFL (1994). Functional role of type I and type II interferons in antiviral defense. *Science*.

[49] Lin R, Genin P, Mamane Y, Hiscott J (2000). Selective DNA binding and association with the CREB binding protein coactivator contribute to differential activation of alpha/beta interferon genes by interferon regulatory factors 3 and 7. *Molecular and Cellular Biology*.

[50] Rasschaert J, Ladrière L, Urbain M (2005). Toll-like receptor 3 and STAT-1 contribute to double-stranded RNA+ interferon-*γ*-induced apoptosis in primary pancreatic *β*-cells. *The Journal of Biological Chemistry*.

[51] Sobel DO, Ewel CH, Zeligs B (1994). Poly I:C induction of *α*-interferon in the diabetes-prone BB and normal Wistar rats. Dose-response relationships. *Diabetes*.

[52] Devendra D, Jasinski J, Melanitou E (2005). Interferon-*α* as a mediator of polyinosinic: polycytidylic acid-induced type 1 diabetes. *Diabetes*.

[53] Flodstrom M, Maday A, Balakrishna D, Cleary MM, Yoshimura A, Sarvetnick N (2002). Target cell defense prevents the development of diabetes after viral infection. *Nature Immunology*.

[54] Sadeharju K, Lönnrot M, Kimpimaäki T (2001). Enterovirus antibody levels during the first two years of life in prediabetic autoantibody-positive children. *Diabetologia*.

[55] Tessier MC, Qu HQ, Frechette R (2006). Type 1 diabetes and the OAS gene cluster: association with splicing polymorphism or haplotype?. *Journal of Medical Genetics*.

